# Identification and evaluation of the novel immunodominant antigen Rv2351c from *Mycobacterium tuberculosis*

**DOI:** 10.1038/emi.2017.34

**Published:** 2017-06-07

**Authors:** Xuezhi Wang, Shuangshuang Chen, Yongjuan Xu, Huajun Zheng, Tongyang Xiao, Yuqing Li, Xing Chen, Mingxiang Huang, Haifeng Zhang, Xijing Fang, Yi Jiang, Machao Li, Haican Liu, Kanglin Wan

**Affiliations:** 1 State Key Laboratory for Infectious Diseases Prevention and Control, Collaborative Innovation Center for Diagnosis and Treatment of Infectious Diseases, National Institute for Communicable Disease Control and Prevention, Chinese Center for Disease Control and Prevention, Beijing 102206, China; 2 Central Laboratory, Beijing Research Institute for Tuberculosis Control, Beijing 100035, China; 3 Prevention and Health Care, Huilongguan Community Health Service Center, Beijing 100096, China; 4 Shanghai MOST Key Laboratory of Health and Disease Genomics, Chinese National Human Genome Center at Shanghai, Shanghai 201203, China; 5 Epidemiology and Health Statistics, School of Public Health, University of South China, Hengyang 421001, Hunan Province, China; 6 Clinical Laboratory, Fuzhou Pulmonary Hospital, Fuzhou 350008, Fujian Province, China; 7 Research and Development Department, Wuhan Institute of Biological Products Co., Ltd, Wuhan 430207, Hubei Province, China

**Keywords:** cytokine, immunogenicity, *Mycobacterium tuberculosis*, Rv2351c, vaccine

## Abstract

There is an urgent need for new immunodominant antigens to improve the diagnosis of tuberculosis (TB) and the efficacy of the TB vaccine to control the disease worldwide. In this study, we evaluated the diagnostic potential of a novel *Mycobacterium tuberculosis* (MTB)-specific antigen, Rv2351c, from region of difference (RD) 7 of the MTB genome, and investigated the potency of the vaccine by identifying its immunological function in human and animal immunological experiments. Twenty T-cell epitopes were identified using TEpredict and prediction tools from the Immune Epitope Database and Analysis Resource. A total of 159 subjects, including 61 patients with pulmonary TB, 38 patients with no TB and 55 healthy donors, were recruited and analyzed with an enzyme-linked immunospot (ELISpot) assay. The ELISpot assay using Rv2351c to detect TB infection, as compared with bacteriological tests as the gold standard, had a sensitivity and specificity of 61.4% (35/57) and 91.4% (85/93), respectively. The ELISpot assay using Rv2351c had a good conformance (*κ*=0.554) as compared with the bacteriological test. Rv2351c also elicited a potent cellular immune response with a high expression of cytokines (IFN-γ (4978±596.7 μg/mL) and IL-4 (68.3±15.5 μg/mL)) and a potent humoral immune response with a high concentration of IgG (1:2.2 × 10^6^), IgG1 (1:4.5 × 10^5^) and IgG2a (1:1.6 × 10^6^) in immunized BALB/c mice. In addition, the ratio of IgG2a/IgG1 indicated that Rv2351c induced cellular immunity in the mice. The results of this study indicated that Rv2351c is an antigen with good immunogenicity that may potentially be used to develop diagnostic techniques and new TB vaccines.

## INTRODUCTION

Despite the widespread use of the Bacille Calmette-Guerin (BCG) vaccine, tuberculosis (TB) continues to be a burden to public health. One-third of the world’s population is asymptomatically infected with *Mycobacterium tuberculosis* (MTB), and 10.4 million new TB cases, 1.4 million deaths from TB and an additional 0.4 million deaths resulting from TB among patients with HIV were reported in 2015 worldwide.^1^ The increased rate of multidrug-resistant and extensively drug-resistant TB and co-infection with HIV/AIDS has worsened the TB burden. Therefore, the rapid diagnosis of latent and active TB and early treatment are the key steps to decreasing TB prevalence. In addition to the development of rapid diagnostic techniques for TB, novel and effective vaccines that may provide long-lasting protection or immunological therapy for individuals immunized with BCG are crucial for controlling and eliminating TB globally.

Because MTB is an intracellular bacterial pathogen surviving and proliferating within the macrophage, both protection and pathogenesis are mediated primarily by cellular responses, which involve interactions of lymphocytes (mainly T cells) and monocyte/macrophage lineages.^2^ Protective immunity against TB is mediated by Th1 CD4^+^ and effector CD8^+^ T cells.^3^ Currently, the identification of MTB-specific antigens is mainly focused on region of difference (RD) genes that are absent from the BCG strains.^4^ Detection based on T-cell immune responses against early secreted antigenic target 6 (ESAT-6) and culture filtrate protein 10 (CFP-10) from RD1 of MTB has been proposed as a tool for the diagnosis of TB infection and used in an enzyme-linked immunospot (ELISpot) assay.^5, 6^ Many other antigens, such as EspC (Rv3615c), MPT-64(Rv1980c), TB7.7 (Rv2654c), HspX (Rv2031c) and EsxJ (Rv1038c), have been identified as immunodiagnostic, but they still cannot meet clinical needs.^7, 8, 9, 10^ Phospholipases are important virulence factors in many bacteria, including *Pseudomonas aeruginosa*,^11, 12^ *Listeria monocytogenes*,^13^ *Clostridium perfringens*^14^ and MTB.^15^ The genome of MTB H37Rv contains three contiguous genes, *plcA*, *plcB* and *plcC*, which are similar to the phospholipase C (plc) gene from *P. aeruginosa*. PlcA (512 aa) is a membrane-associated phospholipase C1 that was originally identified by mass spectrometry of MTB H37Rv^16^ and is encoded by Rv2351c; the expression of the native plcA has been demonstrated through immunoblotting of MTB.^17^ PlcA is essential in the pathogenic MTB complex and is found in both MTB and *M. africanum*, but is absent in all the BCG strains.^18^ Because plcA is an antigen located in RD7 of MTB, we evaluated its potential in the diagnosis of TB infection and the development of TB vaccine. In recent years, many *in silico* epitope prediction tools have been applied to successfully predict epitopes in bacteria and viruses and have facilitated the progress of vaccine development.^19, 20, 21^ The quality of predictions can be improved by combining multiple approaches.^22^ TEpredict is an efficient epitope prediction tool that can predict the interaction between oligopeptides and the transporters associated with antigen processing and can estimate the coverage of peptides in the population on the basis of data on the HLA allele genotypic frequencies.^23^ The Immune Epitope Database and Analysis Resource (IEDB-AR) is a database of experimentally characterized immune epitopes, including T-cell and B-cell epitopes of humans, non-human primates (chimpanzee, gorilla and macaque), rodents (mice and rats) and other species. In this study, to improve the accuracy and efficiency of epitope prediction, we utilized TEpredict and T-cell epitope prediction tools at the IEDB-AR (http://tools.iedb.org/mhci/) to predict the existing linear T-cell epitopes in Rv2351c, because T-cell responses are the basis of the diagnosis of TB infection and the development of TB vaccines. Finally, 20 common T-cell epitopes of Rv2351c were obtained from both TEpredict and IEDB (Table 1), and the results indicated that Rv2351c might have the potential to elicit a T-cell response in patients with TB. Therefore, we constructed a recombinant vector, pET32a-Rv2351c, purified the recombinant Rv2351c protein, and evaluated the diagnostic potential and immunogenicity of Rv2351c in human and animal immunological experiments. Ag85B is an immunodominant antigen that elicits a strong Th1 immune response against MTB challenge and an increased humoral IgG antibody production;^24, 25, 26, 27^ hence, the Ag85B antigen was used as a positive control in animal experiments.

## MATERIALS AND METHODS

### T-cell epitope prediction

T-cell epitopes were identified using TEpredict and other prediction tools from the IEDB-AR. The FASTA format of the amino-acid sequence of Rv2351c has been submitted to TEpredict and IEDB-AR (http://tools.iedb.org/mhci/). The human major histocompatibility complex (MHC) HLA-A and HLA-B alleles were used for the MHC class I antigen peptide binding. The binding affinity for the human MHC alleles that were obtained from prediction tools at IEDB-AR used the half-maximal inhibitory concentration of a biological substance (IC_50_) as the unit of measure. An IC_50_<50 nM indicates high affinity; IC_50_<500 nM indicates medium affinity; and IC_50_<5000 nM indicates low-affinity binding. A lower IC_50_ indicates a stronger binding affinity to the host MHC. The negative logarithm of IC_50_, pIC_50_, was used by TEpredict as the unit of measure to describe the strength of the binding affinity between the peptides and MHC molecules. A pIC_50_<6.3 (IC_50_>500 nM) indicates a low-binding affinity, a pIC_50_ in the range from 6.3 to 7.3 (50 nM<IC_50_<500 nM) indicates a medium binding affinity, and a pIC_50_>7.3 (IC_50_<50 nM) indicates high-binding affinity. Nine-mer MHC class I T-cell epitope prediction was performed using the consensus method. Every epitope was predicted with an immunogenicity score using the T-cell pMHC class I immunogenicity predictor (http://tools.immuneepitope.org/immunogenicity/).

### Construction of the recombinant plasmid pET-32a-Rv2351c

A fragment of Rv2351c was amplified from MTB H37Rv DNA by the polymerase chain reaction (PCR) using the following two primers: (forward): 5′-GCG CGG ATC CAT GTC ACG TCG AGA GTT TTTG-3′, (reverse): 5′-ATATAAG CTT TCA GCT GCA CAG CCC GCT GG-3′.

The PCR was performed in a 25-μL solution containing 1 μL DNA, 1 U Ex Taq HS (Takara Biomedical Technology, Beijing, China), 1 μL forward primer, 1 μL reverse primer, 2.5 μL 10 × Ex Taq buffer and 8.5 μL ddH_2_O. The PCR procedure consisted of an initial denaturation at 94 °C for 5 min, followed by 30 cycles of denaturation at 94 °C for 3 s, annealing at 60 °C for 1 min and extension at 72 °C for 1 min, and a final extension at 72 °C for 5 min. The PCR amplicons were purified using a DNA purification kit (TIANGEN, Beijing, China). The fragment was then cloned into a pET-32a vector after digestion with *Bam*HI and *Hin*dIII, and the recombinant plasmid was transformed into *Escherichia coli* DH5α cells. The recombinant plasmid pET-32a-Rv2351c was isolated from the *E. coli* DH5α cells and chemically transformed into *E. coli* BL21(DE3) cells after the fragment’s identity was confirmed by endonuclease restriction digestion and DNA sequencing.

### Expression and purification of the recombinant Rv2351c protein

The *E. coli* BL21(DE3) cells with the recombinant plasmid were cultured in Luria–Bertani medium overnight at 37 °C. When *OD*_600_ was in the range of 0.6–0.8, isopropyl β-D-1-thiogalactopyranoside was then added to the Luria–Bertani medium to a concentration of 1.0 mmol/L, and the culture was incubated for 3 h at 37 °C. The cells were then collected by centrifugation at 8000*g* for 20 min, and the supernatant and cell pellet were analyzed using 12% sodium dodecyl sulfate-polyacrylamide gels after the cells were processed by ultrasonication. The sodium dodecyl sulfate-polyacrylamide gel electrophoresis (SDS-PAGE) was carried out using 1.5-mm thick 10.1 cm × 7.3 cm glass plates, and the electrophoresis was performed for 30 min at 80 V until the tracer dye reached the end of the gel. The proteins were visualized by staining with Coomassie blue. The results of the SDS-PAGE indicated that the Rv2351c protein was expressed in the form of inclusion bodies. The protein inclusion bodies were washed twice with Tris-HCl buffer containing 1 M NaCl, 2 M urea and 0.5% Triton X-100 and then dissolved in binding buffer (8 M urea, 0.5 M NaCl, 20 mM Tris-HCl and 5 mM imidazole).

The recombinant Rv2351c protein was purified using nickel column chromatography, and the purified lysate was loaded onto a 5- mL Ni-NTA column (His Trap HP, GE Life Sciences, Pittsburgh, PA, USA). The column was washed with wash buffer (8 M urea, 0.5 M NaCl, 20 mM Tris-HCl and 60 mM imidazole), the protein was eluted with elution buffer (8 M urea, 0.5 mM NaCl, 20 mM Tris-HCl and 1 M imidazole) and the column was then stripped with stripping buffer (8 M urea, 0.5 M NaCl, 20 mM Tris-HCl and 10 mM EDTA). The fractions that contained the Rv2351c protein were pooled and dialyzed in phosphate buffer containing 0.2 mM EDTA, 0.9 mM GSH, 0.18 mM GSSG and different concentrations of urea (6, 4, 2, 1 and 0.5 M, and no urea). The refolded protein was concentrated to 1 mg/mL after being analyzed using a BCA protein assay kit (Thermo, Waltham, MA, USA). The purified Rv2351c protein was analyzed by SDS-PAGE and Western blot.

### Study subjects

To evaluate the diagnostic potential of the Rv2351c protein, 159 eligible subjects including patients with and without TB from Fujian and healthy donors from Beijing were enrolled and subjected to analysis with the ELISpot assay using the Rv2351c protein and T-SPOT.TB, along with clinical, microbiological and radiographical examinations. The criteria for enrollment included the following: (1) The patients with active TB were those with clinical and radiographical features of TB confirmed by sputum smear and sputum culture. (2) The patients with no TB were those with other pulmonary diseases than TB. (3) The healthy donors included those with no clinical TB symptoms, no TB contact history and normal X-rays. The sputum samples from patients with no TB were collected, smeared, subjected to acid-fast staining and cultured on Lowenstein Jensen medium. If the sputum smear and/or the bacterial culture result was positive, then the samples were categorized as bacteriologically positive; if the result was negative, they were categorized as bacteriologically negative.

### IFN-γ ELISpot assays

A diagnostic kit for MTB-specific T cells (ELISpot) (QuanBio, Beijing, China) was used to evaluate the magnitude of the response in each case under Rv2351c stimulation. The peripheral blood mononuclear cells (PBMCs) were isolated by Ficoll–Hypaque density gradient centrifugation from 5 to 10 mL of heparinized peripheral blood obtained from each participant. The number of PBMCs was counted using an automatic hematologic analyzer before the sample was diluted to a density of 2.5 × 10^6^/mL with AIV medium. Next, 100 μL PBMCs (2.5 × 10^5^) were cultured with 100 μL of 20 μg/mL peptide or recombinant protein Rv2351c in a 96-well nitrocellulose plate that was precoated with the anti-IFN-γ monoclonal antibody. A cocktail of peptides, including ESAT-6, CFP-10 and Rv3615c, were provided in the kit. Phytohemagglutinin and AIV medium were added to PBMCs as positive and negative controls, respectively. After incubation at 37 °C and 5% CO_2_ for 20 h, the PBMCs and the stimulators were removed, and the assay was conducted according to the kit manufacturer’s instructions. Finally, the spots were counted and analyzed.

The protocol was performed according to the manufacturer’s instructions. The number of spot-forming cells (SFCs) in the negative control was 0–5, and the response was considered positive when the number of SFCs in the target well minus the number of SFCs in the negative control was ≥6. If the number of SFCs in the negative control was 6–10, the response was considered positive when the number of SFCs in the target well was greater than two times the mean value of the number of SFCs in the negative control. The response was considered invalid when the number of SFCs in the negative control was more than 10 or if the number of SFCs in the positive control was less than 20.

### Animals and immunization protocol

Thirty-six 6-week-old, specific-pathogen-free female BALB/c mice were obtained and raised at the Wuhan Institute of Biological Products Co. Ltd. The mice were randomly divided into six groups (*n*=6). To determine the optimal dose that would elicit better immune responses against MTB infection and decrease the adverse effects, each antigen was used at two doses: 20 μg/mouse (low dose) and 50 μg/mouse (high dose). The groups immunized with 20 μg Rv2351c, 50 μg Rv2351c, 20 μg Ag85B and 50 μg Ag85B were denoted Rv2351c-L, Rv2351c-H, Ag85B-L and Ag85B-H, respectively, whereas two groups immunized with PBS or dimethyl-dioctyldecylammonium bromide (DDA)/poly (I:C) were denoted the PBS and DP groups and were maintained as negative controls. DDA is an adjuvant that elicits a mixed Th1/Th2 immune response.^28^ Poly (I:C), a mismatch synthetic double-stranded RNA, was used as an immunostimulant candidate for vaccines against intracellular pathogens, and it elicits cell-mediated immune responses, especially Th1-type immune responses, with antigen-specific CD4^+^ T-cell proliferation and high titers of antigen-specific IgG and IFN-γ.^29^ The protein/adjuvant preparations were prepared by mixing 2.5 mg/mL DDA with 0.5 mg/mL poly (I:C) with or without 20 or 50 μg of Rv2351c or Ag85B. All antigens were emulsified in 100 μL DDA (2.5 mg/mL) that was mixed with 50 μL poly (I:C) (0.5 mg/mL) in advance. All mice were subcutaneously immunized with the immunogen thrice at 2-week intervals.

### Humoral immunity test

To determine the humoral response elicited by the Rv2351c protein, we measured the titer of Rv2351c-specific IgG, IgG1 and IgG2a, because IgG1 is a Th2-type antibody, and IgG2a is a Th1-type antibody. The ratio of IgG2a/IgG1 was plotted to indicate whether either a Th1 or Th2 profile was induced by the Rv2351c protein.

Blood was collected from the mice by retro-orbital puncture 4 weeks after their last immunization. Then, serum was obtained by centrifugation at 3000 r/min for 10 min. Next, 96-well ELISA plates were coated with Ag85B (2 μg/mL) and Rv2351c (2 μg/mL) in 100 μL 0.05 M sodium carbonate buffer (pH 9.6). The plates were washed five times with washing buffer (PBS containing 0.05% Tween 20) before being blocked with 200 μL PBS containing 2% bovine serum albumin at 37 °C for 2 h. Double-diluted (1:5 × 10^3^, 1:1 × 10^4^, 1:2 × 10^4^, 1:4 × 10^4^, 1:8 × 10^4^, 1:1.6 × 10^5^, 1:3.2 × 10^5^, 1:6.4 × 10^5^, 1:1.28 × 10^6^ and 1:2.56 × 10^6^) serum samples were added to the plates (100 μL/well) in duplicate and incubated for 1 h at 37 °C. The samples were incubated with horseradish peroxidase-conjugated goat anti-mouse IgG, IgG1 and IgG2a (Southern Biotech, Birmingham, England) for 1 h at 37 °C after the washing step. Next, 100 μL TMB (Sigma, St Louis, MO, USA) was added, and the samples were incubated for 15 min at 37 °C. The reaction was stopped by adding 50 μL 2 mol/L H_2_SO_4_, and the absorbance was measured at 450 nm. The mean absorbance of the diluted (1:100) negative mouse serum plus 3 SD was noted as the cutoff absorbance for determining antibody titers.

### Cellular immunity test

Four weeks after the last immunization, the mice were killed by cervical dislocation, and the spleens were aseptically removed from the mice. The spleens were ground and passed through a cell strainer. The cell suspensions were concentrated by centrifugation at 1000 r/min for 5 min, and the erythrocytes were lysed using ACK lysis buffer. The erythrocytes were removed, and the splenocytes were washed twice with RPMI-1640 (Gibco, Waltham, MA, USA) medium and then diluted to 1 × 10^6^ cells/mL in RPMI-1640 medium supplemented with 10% FBS and 100 U/mL penicillin–streptomycin. Next, 0.5 mL of splenocytes (1 × 10^6^) were seeded in duplicate in 24-well tissue culture plates (Corning, New York, NY, USA); the cells were stimulated with 0.5 mL of purified Rv2351c protein (5 μg/mL) and Ag85B protein (5 μg/mL) and cells that were stimulated with phytohemagglutinin (1 μg/mL) and AIV medium served as the positive and negative controls, respectively, and were grown at 37 °C with 5% CO_2_ for 72 h. The culture supernatants were collected, and the concentrations of IFN-γ, IL-2 and IL-4 in the supernatants were determined using the cytokine ELISA kit (BD Biosciences, NJ, USA).

### Statistical analysis

The diagnostic performance of Rv2351c was evaluated on the basis of the assay’s sensitivity and specificity. The *χ*^2^-test was used to evaluate the differences between the methods. The coincidence rate between results was analyzed using Cohen’s kappa coefficients. According to the rules of Landis and Koch, *κ*<0.4 indicates poor agreement, *κ*≥0.4 indicates fair to good agreement and *κ*≥0.75 indicates excellent agreement.

The experimental data of cytokine secretion were expressed as the mean±SD. The data were analyzed using the IBM SPSS statistical software package (version 21.0; IBM Corp, Armonk, NY, USA). The *t*-test was used to evaluate the difference between the cytokine level and the antibody titer. *P*<0.05 and *P*<0.001 were considered significant and highly significant differences, respectively, between the experimental groups.

## RESULTS

### T-cell epitope prediction

Rv2351c was predicted to have many medium-affinity (IC_50_<500 nM or pIC_50_: 6.3–7.3) and a small number of high-affinity epitopes (IC_50_<50 nM or pIC_50_>7.3), on the basis of IEDB-AR and TEpredict (data not shown). Twenty overlapping nine-mer T-cell epitopes were identified by TEpredict and IEDB-AR (Table 1). Peptides with a higher score and more HLA alleles to bind were more likely to be immunogenic. As shown in Table 1, peptides 2 and 7 were more likely to be human T-cell epitopes and to elicit a T-cell response in humans.

### Expression and purification of recombinant Rv2351c protein

**Figure 1:**
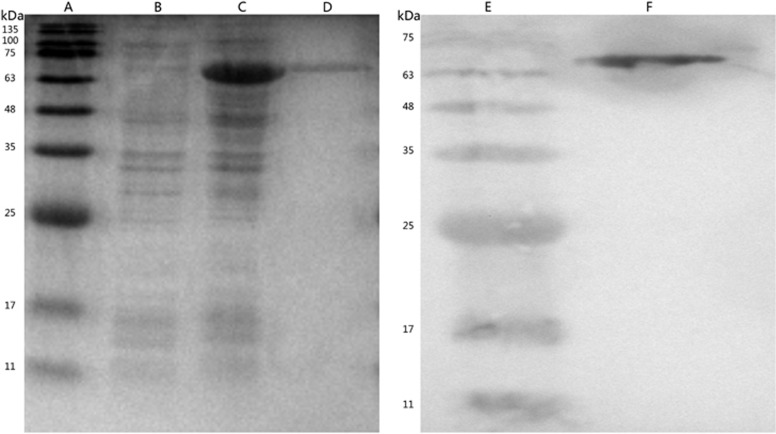
SDS-PAGE and Western blot analysis to detect the purified recombinant Rv2351c protein expression. Lanes: A/E, Standard protein marker; B, non-induced pET-32a-Rv2351c; C, induced pET-32a-Rv2351c; and D/F, purified recombinant Rv2351c protein.

A 1539-bp fragment was successfully inserted into the pET32a vector (Solarbio, Beijing, China) and confirmed by DNA sequencing. As shown in Figure 1, the result of the SDS-PAGE analysis indicated that the Rv2351c was expressed in the form of inclusion bodies (Figure 1C) and was purified as an approximately 69.35 kD recombinant protein (Figure 1F). Western blot analysis was performed using an anti-His antibody to specifically confirm the presence of the recombinant Rv2351c protein (Figure 1F).

### Characteristics of the subjects

From 15 August 2015 to 21 September 2015, a total of 159 subjects including 61 patients with pulmonary TB and 38 patients with no TB were recruited from the Fuzhou Pulmonary Hospital, Fujian, and 60 healthy donors were recruited from the Chinese Center for Disease Control and Prevention, Beijing, China. A total of 154 subjects with valid results and diagnostic information were enrolled for the statistical analyses, whereas five healthy donors were excluded because of invalid ELISpot results in which the number of SFCs in the positive control was less than 20. All the subjects were vaccinated with BCG. The patients in the TB group included microbiologically positive subjects with clinically diagnosed pulmonary TB. The 38 patients with no TB included patients with diseases, such as chronic obstructive pulmonary disease, pneumothorax, pneumonia, bronchitis and lung abscess. The 55 healthy donors showed normal chest X-rays. The 38 patients with no TB and the 55 healthy donors were considered negative controls. The baseline information for the 159 participants is given in Table 2. There were no significant differences in the sex ratio among the three groups (*P*>0.05). The median age of healthy donors was significantly lower than that of the patients with no TB and those with pulmonary TB (Table 2).

### Diagnostic performance of the Rv2351c protein

**Figure 2:**
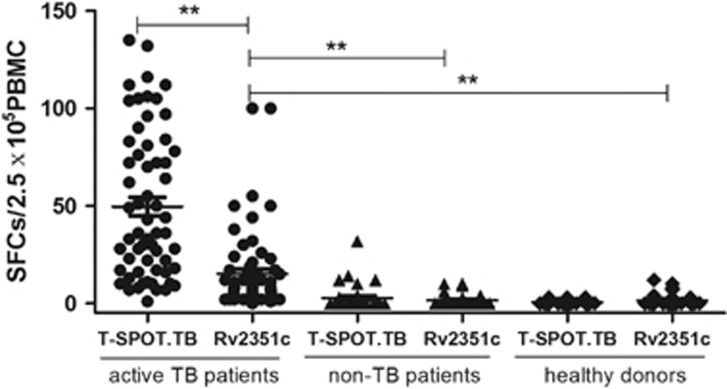
Response magnitude of different subjects against cocktail peptides and Rv2351c in the ELISpot assay. Sixty-one patients with active TB, 38 patients with no TB and 55 healthy donors were enrolled to evaluate the T-cell response to cocktail peptides (Rv3615c, ESAT-6 and CFP-10) and the Rv2351c protein. Responses against the cocktail peptides and the Rv2351c protein were obtained through the T-SPOT.TB assay. The dots represent the response in each case under stimulation with cocktail peptides and Rv2351c. The thick line represents the average response of each group. *P* was calculated by *t*-test to evaluate the statistically significant differences (*P<0.05; **P<0.001).

To evaluate the diagnostic performance of Rv2351c, PBMCs from patients with active TB, patients with no TB and healthy donors were stimulated with the Rv2351c protein for 20 h. Among the 61 patients with clinically diagnosed TB, 37 were positive for Rv2351c; among the 38 patients with no TB and the 55 healthy donors, 4 from each group were positive for Rv2351c. Among the 61 patients with TB, 57 were bacteriologically positive, and all the patients with no TB and healthy donors were bacteriologically negative; therefore, the bacteriological tests could be considered the ‘gold standard’ the sensitivity and specificity of Rv2351c detection by the T-SPOT.TB test were 61.4% (35/57) and 98.2% (56/57), respectively. There was a significant difference between the results of the T-SPOT.TB and ELISpot assays using Rv2351c (*P*<0.05) (Table 3). However, the results of the ELISpot assay using Rv2351c had a moderate overall agreement (78%) with the results of the T-SPOT.TB test using the 3Ag peptide cocktail (Rv3615c, ESAT-6 and CFP-10). Subsequently, the response magnitude of different groups of patients against the cocktail peptides and Rv2351c was analyzed with the T-SPOT.TB assay (Figure 2). The results revealed that the magnitude of the response against Rv2351c in patients with active TB was significantly lower than that against cocktail peptides (*P*<0.01), whereas the magnitude of response of the patients with no TB and the healthy donors against the cocktail peptides and Rv2351c was significantly lower than that of patients with active TB. No significant differences were observed between patients with no TB and healthy donors, thus indicating that the immune responses against MTB did not differ between patients with different pulmonary diseases and healthy donors. This result also indicated that the difference in median age among the three groups had no influence on the ELISpot results. TB infection, but not age, influenced the T-cell response among patients with active TB, patients with no TB and healthy donors.

Collectively, the Rv2351c protein distinguish patients with active TB from those with no TB and healthy donors. The ELISpot assay results, after the samples were stimulated with the Rv2351c protein, also showed good agreement with the gold standard results and a moderate overall agreement (78%) with the results of T-SPOT.TB assay

### Humoral immunity

To determine the ability of Rv2351c to elicit a humoral immune response in BALB/c mice, the level of Ag85B/Rv2351c-specific IgG and the levels of the isotypes IgG1 and IgG2a were tested in the serum using ELISA when the mice were killed four weeks after their last immunization.

**Figure 3:**
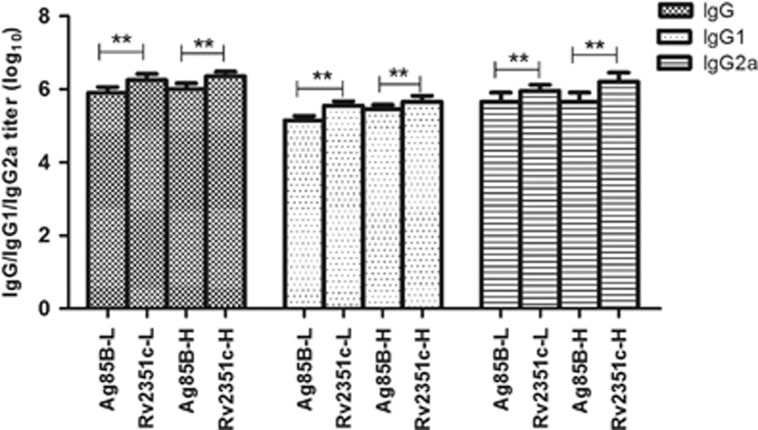
Antibody response against Ag85B and Rv2351c in BALB/c mice immunized with Ag85B or Rv2351c conjugate in DDA/poly (I:C) adjuvant. Serum samples were analyzed for the presence of anti-Ag85B and anti-Rv2351c antibodies via ELISA. The isotype profile of the antibodies was characterized using conjugated secondary antibodies specific for IgG, IgG1 and IgG2a. The data are plotted as geometric mean±SD log_10_ end point titer. *P* was calculated by *t*-test to evaluate the statistically significant differences (*P<0.05; **P<0.001).

As shown in Figure 3, a significantly higher titer of Rv2351c-specific and Ag85B-specific IgG, IgG1 and IgG2a (Figure 3) was observed in the Rv2351c-L (*P*<0.001), Rv2351c-H (*P*<0.001), Ag85B-L (*P*<0.001) and Ag85B-H (*P*<0.001) groups, whereas no IgG/IgG1/IgG2a was detected in the negative control groups (PBS and DP groups). A significantly higher titer of IgG (*P*<0.001) was found in the groups immunized with Rv2351c at a dose similar to that of Ag85B.

The IgG2a/IgG1 ratio in the Rv2351c-L, Rv2351c-H, Ag85B-L and Ag85B-H groups was 2.5, 3.5, 3.1 and 2.2, respectively, thus suggesting a moderate extent of Th1-type immune response to Rv2351c and Ag85B.

As a result, Rv2351c, rather than Ag85B, elicits potent humoral and Th1-type immune responses that are crucial to the control of MTB infection.

### Cellular immunity

**Figure 4:**
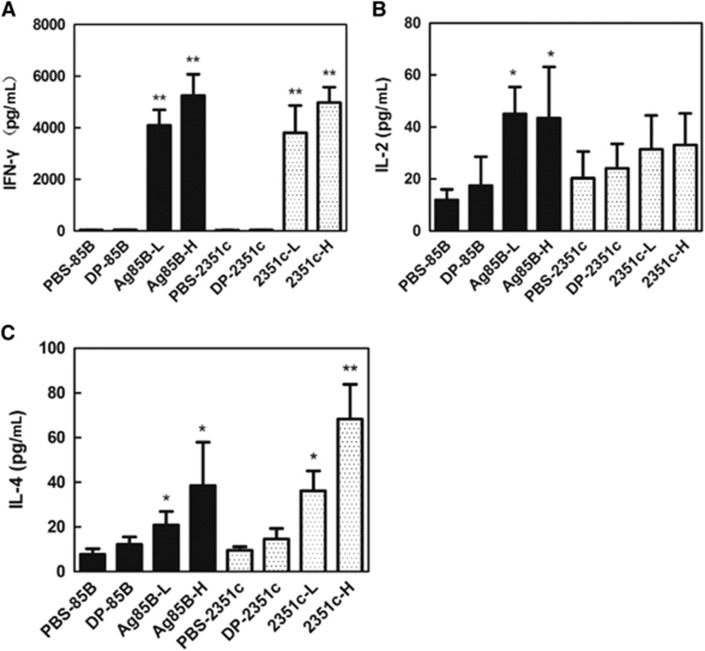
Evaluation of cytokine secretion by splenocytes from immunized mice, isolated and co-cultured with Ag85B or Rv2351c. The splenocytes were prepared four weeks after the mice were immunized with Ag85B or Rv2351c (three times, 2-week intervals). The splenocytes (1 × 10^6^) were then co-cultured with Ag85B (5 μg/mL) or Rv2351c (5 μg/mL) for 72 h before the levels of the cytokines (**A**) IFN-γ, (**B**) IL-2 and (**C**) IL-4 were measured in the culture supernatants using commercial ELISA kits. The data for cytokine secretion are presented as the mean±SD of two independent experiments. The level of statistical significance for differences between the negative control groups and the Ag85B or Rv2351c groups was determined using the *t*-test (*P<0.05; **P<0.001).

To characterize the cellular immune responses elicited by Rv2351c and Ag85B, the concentration of IFN-γ, IL-2 and IL-4 was determined in culture supernatants of splenocytes that were isolated from immunized mice stimulated with Ag85B or Rv2351c. A high concentration of IFN-γ (Figure 4A) was observed in all the groups except the PBS and DP groups. A significantly high production of IFN-γ was observed in the Rv2351c-H (*P*<0.001) and Ag85B-H (*P*<0.001) groups compared with that in the Rv2351c-L and Ag85B-L groups. IL-2 is produced by activated CD4^+^ T cells and CD8^+^ T cells and is involved in immune responses in MTB infection. However, the Rv2351c-L and Rv2351c-H groups showed low production of IL-2 (Figure 4B), whereas the Ag85B-L and Ag85B-H groups showed a significantly higher level of IL-2 compared with the PBS and DP groups (Figure 4B).

IL-4 is produced by Th2 cells and aids in directing naive T lymphocytes in Th2 polarization. All the groups except the PBS and DP groups showed high production of IL-4 (Figure 4C). Rv2351c-L and Rv2351c-H (*P*<0.05) induced higher production of IL-4 than did Ag85B-L and Ag85B-H. The Rv2351c elicited a Th1-dominant immune response with IFN-γ:IL-4 ratios of 105.4 and 73.2 in the Rv2351c-L and Rv2351c-H groups, respectively.

In summary, Rv2351c elicited a cellular immune response in BALB/c mice with high concentrations of IFN-γ and IL-4.

## DISCUSSION

TB has high morbidity and mortality rates, thus leading to severe economic losses in both developing and developed countries. Rapid diagnosis and prompt treatment are the best strategies to control TB. However, conventional detection methods, such as acid-fast staining and sputum bacterial culture, show poor sensitivity, thus leading to a failure to detect some patients with active TB; moreover, radiographic tests with low specificity cannot distinguish between TB and other pulmonary diseases.^30^ The tuberculin skin test sometimes displays false-negative and false-positive results and cannot distinguish between MTB infection and BCG vaccination. The ELISpot assay is now increasingly being used for TB diagnosis because of its high specificity and speed. In this study, we focused on the characterization of Rv2351c, which encodes the plcA protein in MTB. We first evaluated its possible antigenic properties by predicting T-cell epitopes with TEpredict and IEDB-AR. The results of the T-cell epitope prediction indicated that Rv2351c may have antigenic properties in humans with 20 predicted HLA_A/HLA_B T-cell epitopes. To verify this possibility, we purified the recombinant Rv2351c protein and investigated the diagnostic performance of Rv2351c in a study cohort including patients with TB, patients with no TB but with other pulmonary diseases and healthy donors. To determine the diagnostic value of Rv2351c, the sensitivity, specificity and kappa value of an assay using Rv2351c were analyzed, and good agreement (*κ*=0.554) was observed between the results of this assay and a bacteriological test; the ELISpot assay using Rv2351c had a moderate overall agreement with the T-SPOT.TB assay using cocktail peptides (Rv3615c, ESAT-6 and CFP-10). Whereas 37 patients with TB were positive after Rv2351c stimulation, 24 individuals remained negative, thus suggesting a variation in the inter-patient response. Four non-TB patients and four healthy donors tested positive in the assay using Rv2351c, and we speculated that this result may have resulted from a cross reaction induced by the Rv2351c protein or a latent infection. However, two of these four patients with no TB tested positive with the T-SPOT.TB assay using Rv2351c, thus indicating that these cases were more likely to have had a latent infection. Previous studies on the genetic polymorphisms in the *plc* genes, including *plcA*, have reported genomic deletions resulting in the loss of parts of genes or complete genes from the plcABC and/or plcD loci in clinical MTB isolates.^31^ Because the exact data regarding Rv2351c deletion among MTB clinical isolates are unavailable, more research on the deletion of Rv2351c in clinical isolates should be undertaken before its use in the diagnosis of TB infection. Given the polymorphism of the *Rv2351c* gene, Rv2351c might not be the best choice, as compared with other conserved genes to be used as a TB diagnostic tool. However, according to our results, its good diagnostic performance in TB infection was clear. Use of a combination of different antigens can improve diagnostic performance in ELISpot assays.^7^ Therefore, we speculated that the diagnostic performance of Rv2351c might also be improved by combining it with other widely used antigens, such as ESAT-6, CFP-10 or Rv3615c. Hence, Rv2351c has the potential to be used as a supplement for other immunodominant antigens.

Because rapid diagnosis increases the treatment duration of TB, developing an effective vaccine that protects against TB is also important to limit the disease. The BCG vaccine is currently the only TB vaccine used in humans that can protect children from severe disseminated diseases, such as tubercular meningitis and hematogenous disseminated TB, but it cannot protect adults against ‘reactivation’ TB later in life.^32^ A TB subunit vaccine has the potential to replace BCG or to be used as a complement to boost BCG-elicited immune responses when it is combined with a recombinant fusion protein that induces effective protective immunity against TB infection.^33, 34, 35, 36^ Presently, increasing attention is being focused on TB subunit protein vaccines, because they are safe, can be used in immunocompromised individuals and their immune reactivity is not influenced by previous exposure to environmental mycobacterium.^37^

More detailed studies on immunoprotection against MTB infection are crucial for effective vaccine development. Undoubtedly, cellular immunity plays a crucial role in the control of MTB infection, because MTB is an intracellular bacterial pathogen that survives and proliferates within macrophages.^2^ Cytokines are also an important factor determining the immune response toward the desired Th1 and Th2 bias. Cytokines, such as IFN-γ, IL-2, IL-12 and TNF-α, which are responsible for cellular immunity against MTB and are produced by Th1 cells, not only activate macrophages, but also promote polarization of effector Th1 cells.^38^ In contrast, IL-4, IL-5, IL-10 and IL-13, which are produced by Th2 cells, are responsible for inhibiting macrophage functions and promoting antibody responses.^39^ IFN-γ is the most important cytokine marker in T-cell stimulation assays and is involved in protective immunity of the host toward mycobacterial antigens, because it activates macrophages in conjunction with TNF-α, thereby facilitating the killing of intracellular mycobacteria. As expected, Rv2351c induced a significantly higher level of specific IFN-γ and IL-4 production, and the IFN-γ/IL-4 ratio indicated a Th1-dominant immune response. Compared with a DNA vaccine of *M. bovis* that is administered with the Ag85B antigen, Rv2351c elicited a more potent cellular immune response with much higher concentrations of IFN-γ and IL-4.^40^

The role of humoral immune responses in MTB infection has been ambiguous. However, much research has indicated that adaptive immune responses contribute to the outcome of MTB infection, because antibodies can affect the interaction between mycobacteria and other components of the immune system.^41^ Therefore, it is important to consider humoral responses, especially given that understanding of the pathogenic mechanisms underlying MTB infection is still insufficient. Many studies have shown that several classical functions, such as opsonization and complement activation, which are mediated by antibodies, play an essential role in the defense against MTB.

Human IgG enhances complement activation and increases the phagocytosis of MTB by macrophages.^42^ In a previous study, increased complement activation by BCG has been observed among patients with TB with a high IgG2 to LAM ratio.^43^ Moreover, IgM, IgG1, IgG3 and IgA have been confirmed to be protective antibodies against MTB. In our study, significantly higher levels of anti-Rv2351c IgG, IgG2a and IgG1 were observed in mice immunized with the Rv2351c protein. The efficacy of antibodies primarily relies on three aspects: the titer of the antibody available, the structural features of the immunoglobulin molecule and the immunological state of the host.^44^ Therefore, Rv2351c, which induces high-level production of IgG, IgG1 and IgG2a, has great potential to protect against MTB. Because cellular and humoral immunities interact with and influence each other, both should be considered in designing new vaccines. The recombinant BCG strain expressing protein from RD1 induced increased protective efficacy, thus suggesting that supplementation of BCG with a subunit Rv2351c vaccine may be a good vaccination strategy.^45^

An optimal adjuvant can be of equal importance as an antigen in vaccine development. Adjuvants not only increase opportunities for the antigen to be recognized by the immune system by inhibiting the antigen’s degradation and extending the time that the antigen exists *in vivo*, but also change the type of immune response elicited by the antigens. Because proteins alone are poorly immunogenic and prone to degradation, adjuvants, such as DDA and poly (I:C), have been considered in animal experiments. Our study indicated that DDA/poly (I:C) are promising adjuvant candidates to supplement the induction of potent cellular and humoral immunity by Rv2351c and Ag85B. However, more attention should be given to the safety and stability of poly (I:C) before its clinical application because it has the potential to induce excessive immune and autoimmune disorders.^46^

In summary, we confirmed that Rv2351c is an immunodiagnostic antigen that can distinguish patients with TB from those without TB and healthy donors. This study also revealed that Rv2351c is an immunodominant antigen that elicits potent cellular and humoral immune responses in Rv2351c-immunized mice. Therefore, Rv2351c has potential for use in the diagnosis of TB and in a subunit vaccine. This study lays a foundation for the application of Rv2351c in TB vaccine development.

## Figures and Tables

**Table 1:** T-cell epitopes predicted by TEpredict and IEDB

**Peptide ID**	**Location (start–end)**	**Amino-acid sequence**	**Immunogenicity score**	**Number of bound HLA alleles**
1	6–14	FLTKLTGAG	−0.1272	2
2	18–26	FLMDWAAPV	0.24439	8
3	46–54	IVLLMQENR	−0.21408	4
4	76–84	FQQMGWNPM	0.01527	6
5	134–142	WLPAQATTR	0.01524	2
6	146–152	YVPLTMGYY	−0.11582	4
7	163–171	YLLADTFTI	0.21539	7
8	169–177	FTICDGYHC	0.05823	4
9	180–188	LTGTLPNRL	0.0536	2
10	236–244	YQNKGLGRF	−0.1377	4
11	281–289	FAADVRANR	0.15318	4
12	295–303	WLVPNILQS	0.04418	2
13	315–323	VSMVTALRI	0.08005	3
14	317–325	MVTALRILL	0.19573	4
15	323–331	ILLSNPAVW	−0.12967	3
16	363–371	FVTVPNIDA	0.16253	2
17	390–398	CIVISPYSR	−0.124	3
18	434–442	VVGDMTSAF	−0.2179	6
19	473–481	VVLGTTDGA	0.14336	2
20	485–493	IPYRVPYPQ	0.07104	6

**Table 2:** Baesline data of the participants enrolled in the study

	**PTB**	**Non-TB**	**HD**	**Total**
*Total gender*	61	38	60	159
Male	43	22	26	91
Female	18	16	34	68
*Age, years*				
Median (±SD)	43.5±18.0	54.3±19.3	24.7±2.0^a,b^	

Abbreviations: pulmonary tuberculosis, PTB; patients with pulmonary disease but not TB, Non-TB; healthy donors, HD; standard deviation, SD.

a *P*<0.05 (median age between healthy donors and PTB group).

b *P*<0.05 (median age between healthy donors and PTB group).

**Table 3:** Comparison of results using the bacteriological test results as ‘gold standard’ for TB diagnosis

	**T-SPOT.TB**	**ELISpot-2351c**	***P*****-value**
Sensitivity	98.2% (56/57)	61.4% (35/57)	0.000
Specificity	94.6% (88/93)	91.4% (85/93)	0.388
Youden index	0.928	0.528	
Kappa value	0.916	0.554	

Note: 57 TB patients were diagnosed positive and 93 were diagnosed negative with the ‘gold standard’.
